# Control method of stepping motor for spaceborne solar irradiance spectrometer

**DOI:** 10.1038/s41598-022-06979-x

**Published:** 2022-02-21

**Authors:** Yan Wang, Jianing Wang, Zhanfeng Li, Yu Huang

**Affiliations:** 1 Changchun Institute of Optics, Fine Mechanics and Physics, Chinese Academy of Sciences, Changchun, 130033 China; 2 University of Chinese Academy of Sciences, Beijing, 101408 China

**Keywords:** Aerospace engineering, Optical techniques

## Abstract

The encoder is not included in the hardware design of the Spaceborne Solar Irradiance Spectrometer, so in this paper, a high precision control method of stepping motor is proposed, which can position accurately without encoder. When the wavelength scanning mechanism is controlled, the validity of the hall signal is judged by controlling the forward and reverse rotation of the motor and the fixed steps of the motor. When the hall signal is valid, the operation of returning to the starting position is performed normally. The motor drive is subdivided into 16 substeps, and each substep is only 0.1125°. The control method of turntable mechanism is to quickly return to the starting position, after overshoot, one-step reverse rotation with appropriate delay to move out of hall effective area, and then identify the starting position. The experimental results show that the method can meet the requirements of wavelength repeatability less than 0.01 nm, wavelength calibration accuracy less than 0.05 nm, and calibration accuracy stability less than 0.2%. At the same time, the volume and weight of the system are reduced, and the miniaturization of the system is realized.

## Introduction

Solar radiation is the most important external energy of the earth's climate system. Monitoring the change of solar radiation is of great significance to solar physics and atmospheric physics. At the same time, the accurate measurement of solar radiation can not only improve the retrieval accuracy of trace gases, but also help to carry out the assessment of climate effects according to different ground features^1^.

In order to meet the requirements of high spectral resolution, high sensitivity and large dynamic range, the scanning grating instrument is usually used in solar spectral monitoring. Typical solar spectrum monitoring instruments include Solar Ultraviolet Spectral Irradiance Monitor (SUSIM)^2–4^, Solar-Stellar Irradiance Comparison Experiment (SOLSTICE)^5–7^, Spectral Irradiance Monitor (SIM)^8–10^, Solar Spectrum (SOLSPEC)^11–13^.

Spaceborne Solar Irradiance Spectrometer (hereinafter referred to as spectrometer) is a high stability, compact scanning spectrometer, which can monitor the changes of solar radiation by tracking and observing the sun. The main moving parts of the spectrometer include wavelength scanning mechanism and turntable mechanism, which use stepping motor as the control mechanism^14^. Under the condition that the mechanical structure meets the requirements, the repeatability and accuracy of the spectrometer directly depend on the control accuracy of the wavelength scanning mechanism and the turntable mechanism.

Stepping motor control is usually divided into open-loop mode and closed-loop mode^15,16^. The advantages of open-loop mode are simple, easy implementation and low cost, but the disadvantage is low precision. The closed-loop mode is suitable for high-precision scenarios, but the introduction of feedback loop increases the complexity of the system. For the spaceborne solar irradiance spectrometer, the closed-loop mode can ensure the accuracy and stability. However, as an actual satellite load, its volume, weight and power consumption are strictly limited, and the closed-loop mode can not be realized in hardware design and structure design. Therefore, the structure of the spectrometer innovatively adopts the design without encoder, which makes it difficult to locate the moving parts. In this paper, an open-loop motor control logic independent of encoder feedback is proposed, which not only ensures the detection accuracy, but also effectively reduces the complexity and difficulty of structural design. The method controls the starting position by identifying the hall signal, and drives the motor by subdividing the number of steps to improve the accuracy. In this design, in order to deal with the possible locked rotor risk of the reciprocating lead screw structure, the multi hall structure is innovatively used with the control logic to realize the accurate return to the starting position at any position. At the same time, the software control logic has the functions of independent troubleshooting and anti locked rotor, which greatly improves the reliability of the spaceborne instrument. The control of the turntable mechanism motor innovatively adopts the method of rapid return to the starting position and overshoot, one-step reverse rotation with appropriate delay is used to remove the hall effective area as the starting position of the turntable mechanism, which ensures that the detection time is not compressed in the process of returning to the starting position, and avoids overshoot and ensures the accuracy. The mercury lamp and deuterium lamp are used as standard light sources to irradiate the spectrometer directly, and the spectral data are obtained by scanning. The spectral response characteristics are judged by the output spectral response curve to check whether the wavelength repeatability, wavelength calibration accuracy and calibration stability meet the index requirements, which proves the effectiveness of the control method proposed in this paper.

## Methods

**Figure 1:**
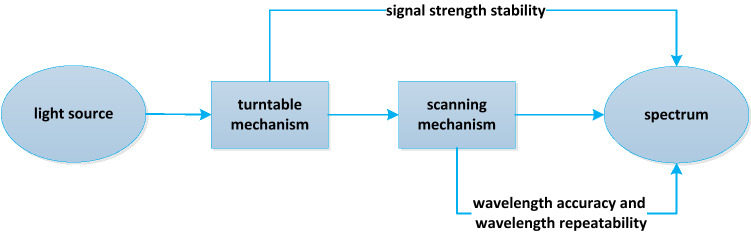
Schematic diagram of influencing factors.

The working principle of solar irradiance spectrometer is that the light irradiates on the diffuse reflector after passing through the baffle and quartz flat window. The light emitted from the diffuse reflection plate enters the incident slit of the monochromator and is scanned by the secondary grating. The monochromatic radiation of different wavelengths is output through the exit slit. Finally, the light signal is received by the detector and converted into electrical signal. A fused silica glass window is added in front of the diffuse reflector through the turntable mechanism to protect the diffuse reflector and optical elements from the decay caused by solar extreme ultraviolet radiation. During the on orbit operation, the turntable mechanism switches according to the working mode, which can protect the optical path and convert different band filters (or attenuators). The scanning mechanism drives a mechanical swing rod to divide the incident wide spectrum light source according to the wave band. The wavelength scanning mechanism converts the rotating motion of the motor into the linear motion of the slider. The spring tightens the slider and the swing rod tightly to realize the synchronous operation without gap. Under the push of the swing rod, the grating rotates around the grating axis, so as to obtain light of different wavelengths at the exit slit. Through calibration, the relationship between the motor steps and the exit wavelength can be obtained. The wavelength scanning mechanism is essentially a sinusoidal mechanism. There is a linear relationship between the motor steps of the wavelength scanning mechanism and the outgoing wavelength. The change of the wavelength scanning mechanism means the change of the outgoing wavelength. Therefore, the positioning repeatability and absolute positioning accuracy of the wavelength scanning mechanism will also directly affect the repeatability and accuracy of the finally obtained spectral data. Several filters are evenly distributed on the turntable mechanism. During detection, the incident light needs to pass through the filter first. The uniformity of the coating layer of the filter has a certain impact on the energy finally entering the optical system. During actual processing, the filter film generally has a certain uniformity error, which requires that the incident light must be incident to the optical system from the same area of the filter every time. If there is a significant error in the positioning repeatability of the turntable mechanism, when the wavelength scanning mechanism scans, the energy received by the detector at the same grating angle fluctuates, and when fitting the central wavelength, the correspondence between the wavelength and the number of motor steps may be slightly offset, resulting in errors in wavelength repeatability. Therefore, the turntable mechanism mainly affects the stability of the signal strength, and the wavelength scanning mechanism mainly affects the wavelength accuracy and wavelength repeatability, and then both affect the quality of the final obtained spectral data, as shown in Fig. 1.

### Control method of wavelength scanning mechanism

**Figure 2:**
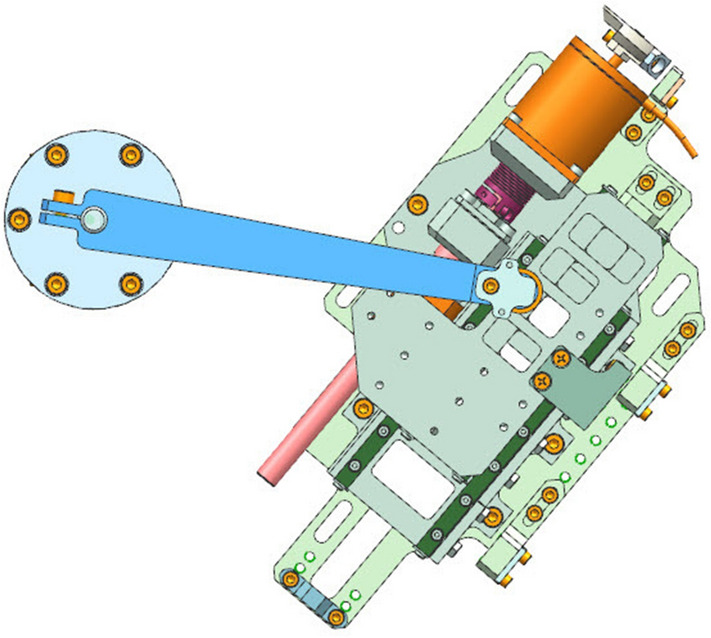
Structure diagram of wavelength scanning mechanism.

Wavelength scanning mechanism is one of the key mechanisms of solar irradiance spectrometer, which determines the wavelength repeatability, wavelength linearity and other technical indicators of the spectrometer. The wavelength scanning mechanism consists of stepping motor, grating shaft, swing bar, spring, slider, roller and screw mechanism, as shown in Fig. 2. When working, the stepping motor drives the lead screw to rotate, and the two guide rails constrain the nut to make a linear motion, so as to push the slider to move along the axis direction of the lead screw.

**Figure 3:**
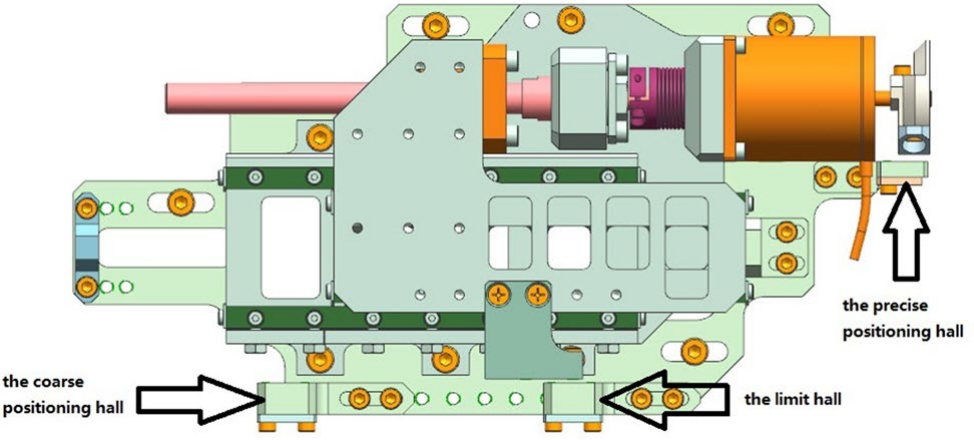
Hall’s position diagram of scanning mechanism.

The wavelength scanning mechanism is a reciprocating motion structure with limited linear length. Considering the design requirements of miniaturization and lightweight of spectrometer, the wavelength scanning mechanism has no feedback signal such as encoder, so it is unable to carry out closed-loop control. Therefore, in order to ensure the repeatability, it is very important to determine the starting position of each scan. In order to determine the starting position, the solar irradiance spectrometer is equipped with three halls for positioning, which are the precise positioning hall, the coarse positioning hall and the limit hall, as shown in Fig. 3. The positioning coarse hall and the limit hall are used to characterize the two ends of the effective stroke of the wavelength scanning mechanism, and the precise positioning hall is used for the accurate positioning of the slider.

**Figure 4:**
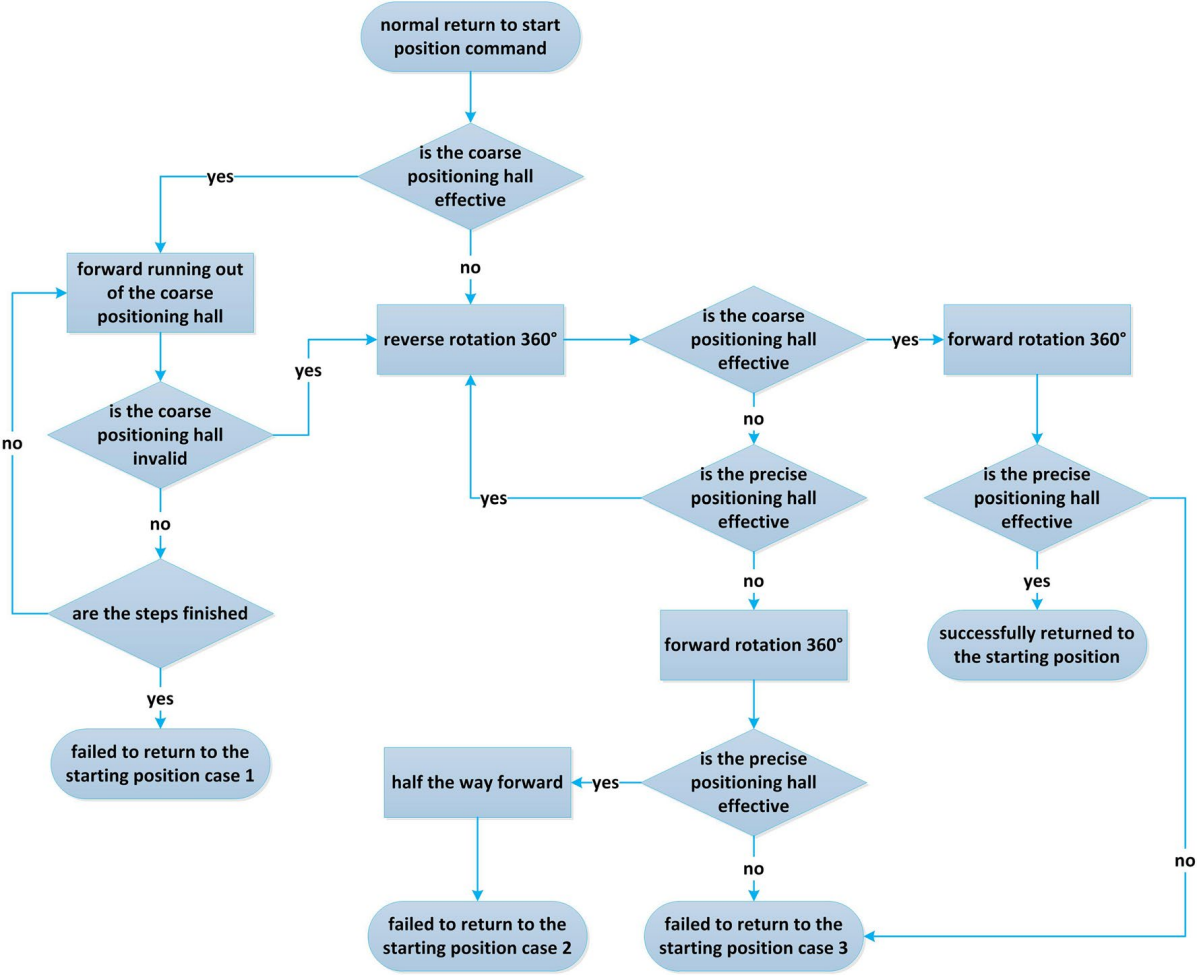
Flow chart of wavelength scanning mechanism returning to starting position.

Before each scan, the lead screw mechanism returns to the starting position. In the process, the validity of the hall signal is judged by controlling the forward and reverse rotation of the motor and the fixed number of steps of the motor operation. The motor drive is subdivided into 16 substeps, and each substep is only 0.1125° to ensure the high precision of the lead screw swing rod spectroscopic structure. The specific control process is shown in Fig. 4.

The flow control method of wavelength scanning mechanism returning to the starting position is shown in Algorithm 1.

Algorithm 1: the control algorithm of wavelength scanning mechanism returning to the starting position normally.

Input: the command to return to the starting position.

Output: status.

Step 1: Judge whether the coarse positioning hall is effective. If not, go to step 5.

Step 2: Run forward to outside the effective area of the coarse positioning hall.

Step 3: Judge whether the coarse positioning hall is effective. If valid, go to step 5.

Step 4: Judge whether the steps are finished. If not, go to step 2. If the steps are finished, go to step 13.

Step 5: Rotate 360° in reverse.

Step 6: Judge whether the coarse positioning hall is effective. If not, go to step 9.

Step 7: Rotate 360° forward.

Step 8: Judge whether the precise positioning hall is effective, jump to step 13.

Step 9: Judge whether the precise positioning hall is effective. If valid, go to step 5.

Step 10: Rotate 360° forward.

Step 11: Judge whether the precise positioning hall is effective. If not, go to step 13.

Step 12: Run forward half a stroke.

Step 13: Output different working states.

After receiving the command to return to the starting position, perform the operation of returning to the starting position according to the steps of Algorithm 1. If the return status indicates failure, the specific failure hardware can be determined according to the specific failure type. The specific meaning of failure information is shown in Table 1.

**Table 1:** Meaning of failure status information.

Serial number	Name	Meaning
1	Failure status case 1	When returning to the starting position normally, the motor stalls and the operation fails
2	Failure status case 2	When returning to the starting position normally, the coarse hall fails and the operation fails
3	Failure status case 3	When returning to the starting position normally, the precise hall fails and the operation fails

Because the selected devices are aerospace grade, it can ensure the stability and reliability of machinery and electronics in the vacuum environment with strong radiation. During scan, the motor operation control is carried out in strict accordance with the sampling interval, without accumulated error.

### Control method of turntable mechanism

**Figure 5:**
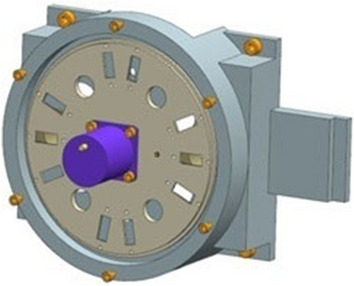
Structure diagram of turntable mechanism.

The turntable mechanism is composed of turntable, stepping motor, turntable base and protective cover. As shown in Fig. 5. According to the requirements of working mode, a certain number of holes are evenly distributed on the turntable, which is equipped with blind plate, protection window, attenuator, filter and so on. When the spectrometer is scanning and detecting, the turntable mechanism moves the quartz window into the optical path, and then according to the needs of different spectral segments, it turns into the window with the filter or attenuator with the level selection. When it is not working, the blind plate moves into the optical path to protect it. The turntable mechanism is equipped with a main hall device, one standby Hall device, both of which are used to determine the starting position. During the detection, the control of the turntable mechanism first runs to the starting position, and then runs to different hole positions according to the working mode.

**Figure 6:**
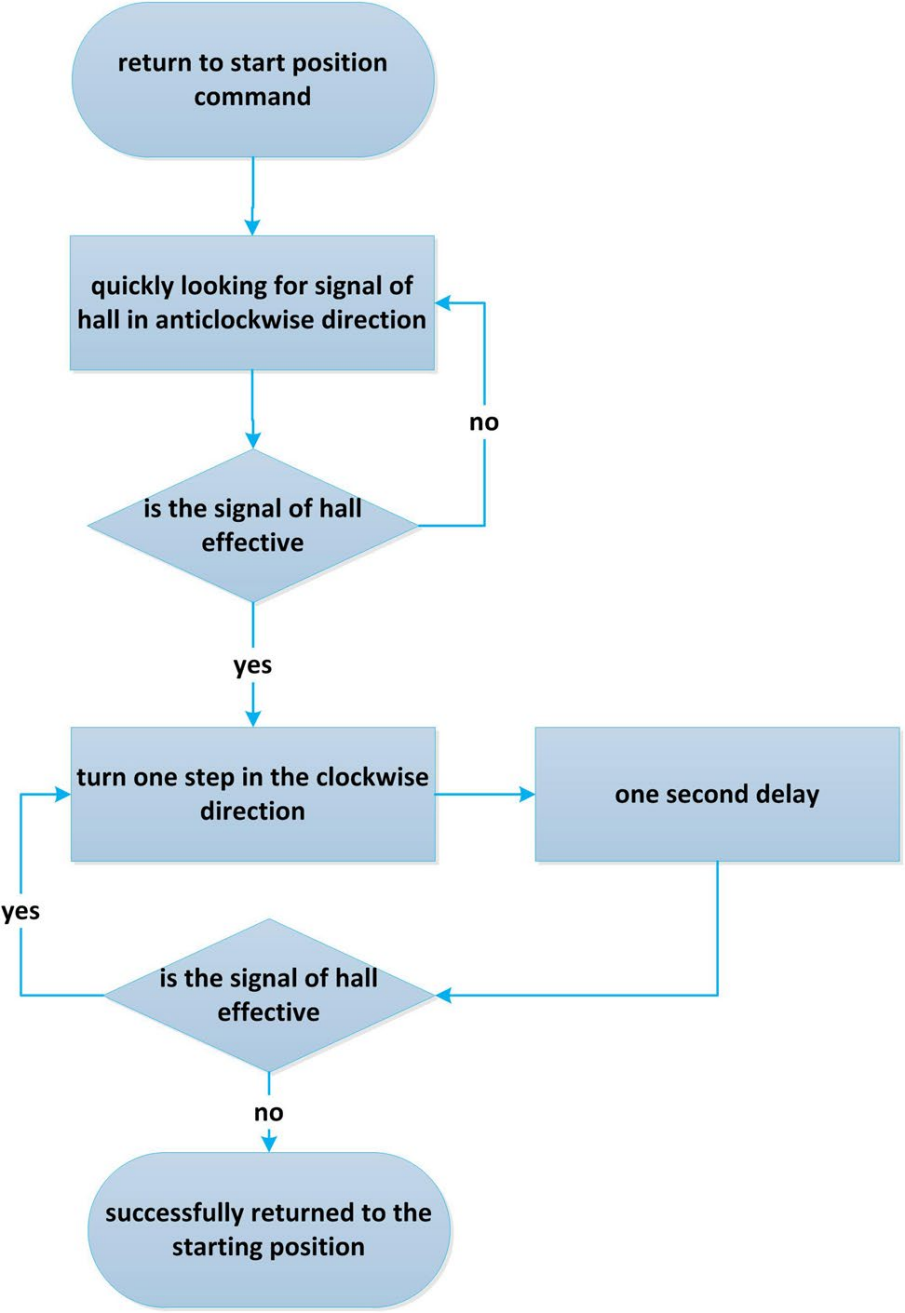
Control flow chart of turntable mechanism returning to the starting position.

The turntable mechanism with filter is the first channel through which the incident light passes. Its rotation speed and accuracy of returning to the starting position directly determine the overall detection accuracy of the spectrometer. It is necessary to realize the high-precision return to the starting position of the turntable mechanism on the premise of reducing the rotation time as much as possible. At this time, the main problem is that the hall signal has microsecond instability in the process of motor rotation. When the motor returns to the starting position quickly, it will produce overshoot due to mechanical inertia. In the design, after fast return to the starting position and overshoot, one-step reverse rotation with appropriate delay is used to remove the hall effective area as the starting position of the turntable mechanism, which ensures that the detection time is not compressed in the process of returning to the starting position, and avoids overshoot and ensures the accuracy. The specific process is shown in Fig. 6.

The flow control method of turntable mechanism returning to the starting position is shown in Algorithm 2.

Algorithm 2: the control algorithm of turntable mechanism returning to the starting position normally.

Input: the command to return to the starting position.

Output: status.

Step 1: Run quickly in the anticlockwise direction to find the hall signal.

Step 2: Judge whether the hall signal is effective. If it is invalid, go to step 1.

Step 3: Control the motor to run in the clockwise direction for 1 step.

Step 4: Delay 1 s.

Step 5: Judge whether the hall signal is effective. If valid, go to step 3.

Step 6: Output the status of successful return to the starting position.

On the hardware, the turntable mechanism is equipped with two hall devices, one is main and the other is standby. If the main hall fails, you can input the switch hall command and use the backup hall to ensure that the turntable mechanism will not have a single point of failure when in orbit, which will affect the work of the spectrometer.

## Results

Because the solar irradiance spectrometer has no encoder and other feedback components, the effectiveness and results of the proposed method can not be visually verified by the feedback information. The on orbit observation target of spaceborne solar irradiance spectrometer is the sun. As a very stable radiation source, the annual change of the visible band and near-infrared band of the sun is only a few thousandths. In order to detect the small change of solar spectral radiation, the spectrometer needs to have high stability, and the wavelength repeatability is an important factor affecting the stability of the spectrometer. Therefore, the wavelength repeatability index of spaceborne solar irradiance spectrometer is required to be 0.01 nm, which poses a high challenge to the control of motor. Due to the limitations of space, weight and power consumption, the spaceborne solar irradiance spectrometer can not realize the closed-loop control with encoder in hardware design and structure design. Therefore, the method proposed in this paper is a high-precision control method of stepping motor without encoder. Although this method is not compared with the test results with encoder, the results show that this method meets the index requirements and can ensure the spectrometer to complete the on orbit detection task. At the same time, the volume and weight of the system are reduced, and the miniaturization of the system is realized. In order to verify the effectiveness of the proposed method, the mercury lamp and deuterium lamp are used as light sources, and the wavelength repeatability and calibration accuracy of the spectrometer are obtained through the mercury lamp spectral calibration results, so as to prove that the control method of the scanning mechanism is effective and meets the technical requirements. The light signal stability of the deuterium lamp is measured several times at the same hole position, and the signal strength stability is calculated. The results prove whether the control method of the turntable mechanism is effective and meets the requirements of technical indicators.

### Spectral calibration

**Figure 7:**
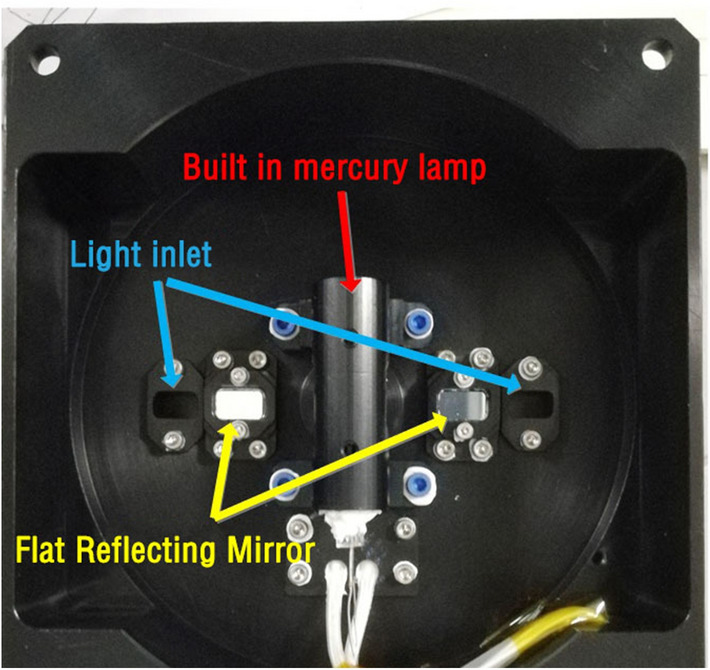
Mercury lamp in solar irradiance spectrometer.

Mercury lamp is a common spectral calibration light source. High pressure mercury lamp has rich atomic emission spectrum, covering the ultraviolet to near infrared band. Its spectral line position does not change with the environmental conditions, and the spectral line width is very small. It can be considered as an ideal spectral line. It has been widely used spectral calibration and calibration of spectrometers in the ground or on orbit. The solar irradiance spectrometer has a built-in mercury lamp, which can irradiate the diffuse reflectors through two flat mirrors, so as to achieve on orbit spectral calibration. As shown in Fig. 7.

**Figure 8:**
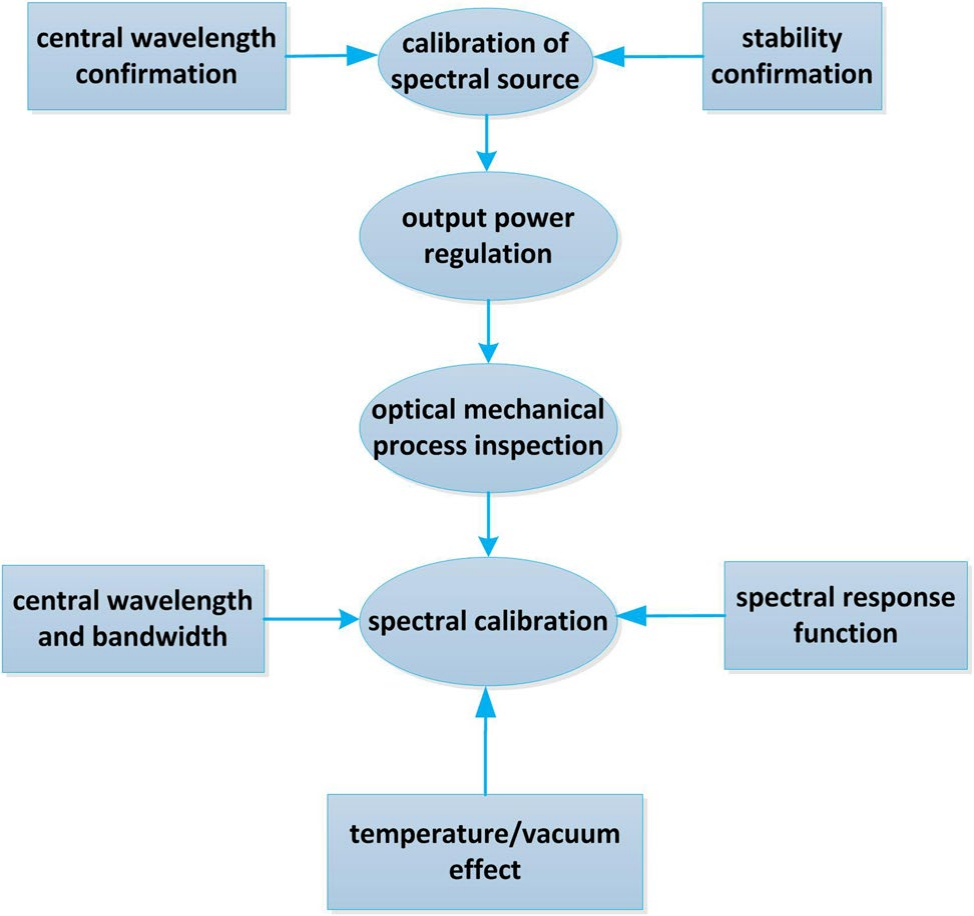
Flow chart of spectral calibration.

In this paper, the built-in mercury lamp of spectrometer is used to test the spectral resolution, wavelength repeatability and wavelength calibration accuracy in vacuum environment, which proves that the control method of stepping motor proposed in this paper is effective and meets the index requirements. The flow of spectral calibration is shown in Fig. 8.

The specific process is as follows.

1. Turn on the mercury lamp inside the spectrometer and preheat it for 20 min;
2. As a standard light source, mercury lamp directly irradiates the entrance pupil of spectrometer;
3. We switched on the wavelength scanning with the scanning interval of 0.1 nm. The characteristic spectral lines of 184.891 nm, 253.652 nm and 296.728 nm were scanned;
4. The same spectral line was scanned 5 times.

The data processing process is as follows.

1. The spectral response curve of each mercury lamp spectrum is drawn from the output data;
2. The center wavelength was obtained by Gaussian fitting. FWHM is the spectral resolution;
3. The standard deviation of multiple measurements of central wavelength is wavelength repeatability, defined as s. s is calculated as follows.
  1 $$s = \sqrt {\frac{{\sum\limits_{k = 1}^{5} {(q_{k} - \mathop q\limits^{ - } )^{2} } }}{5 - 1}}$$
  where $q_{k}$ is the result of the kth measurement and $\mathop q\limits^{ - }$ is the arithmetic mean of the five measurements;
4. The difference between the average value of multiple measurements of the central wavelength and the standard wavelength is the wavelength calibration accuracy.

### Test results of scanning mechanism

**Figure 9:**
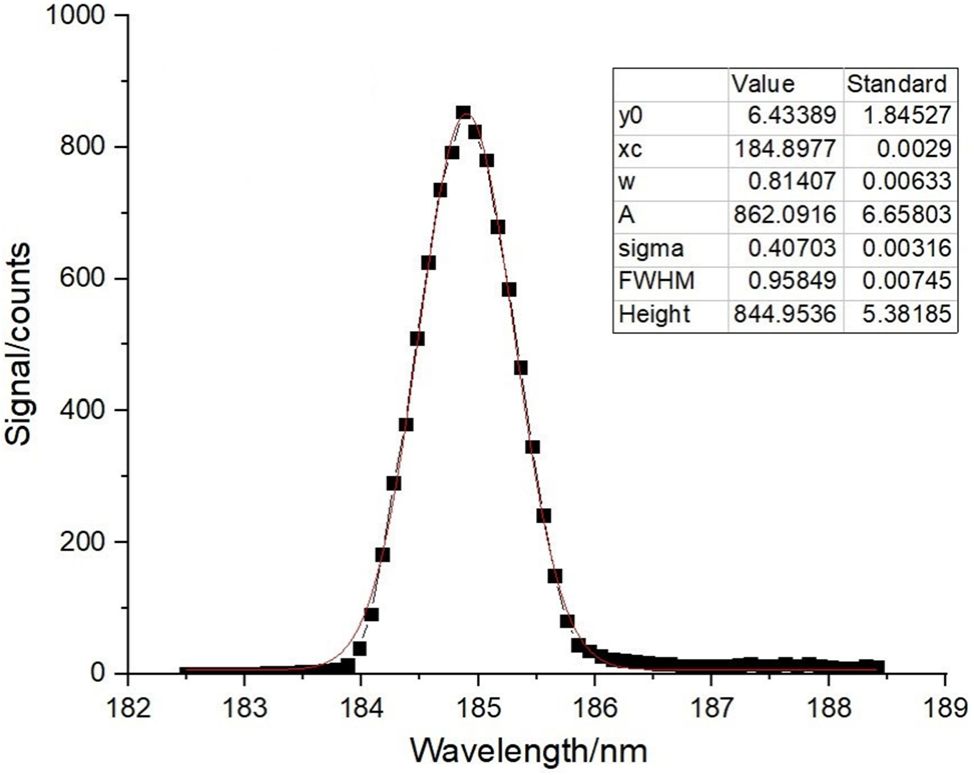
Spectral response and Gaussian fitting curve of mercury lamp at 184 nm.

**Figure 10:**
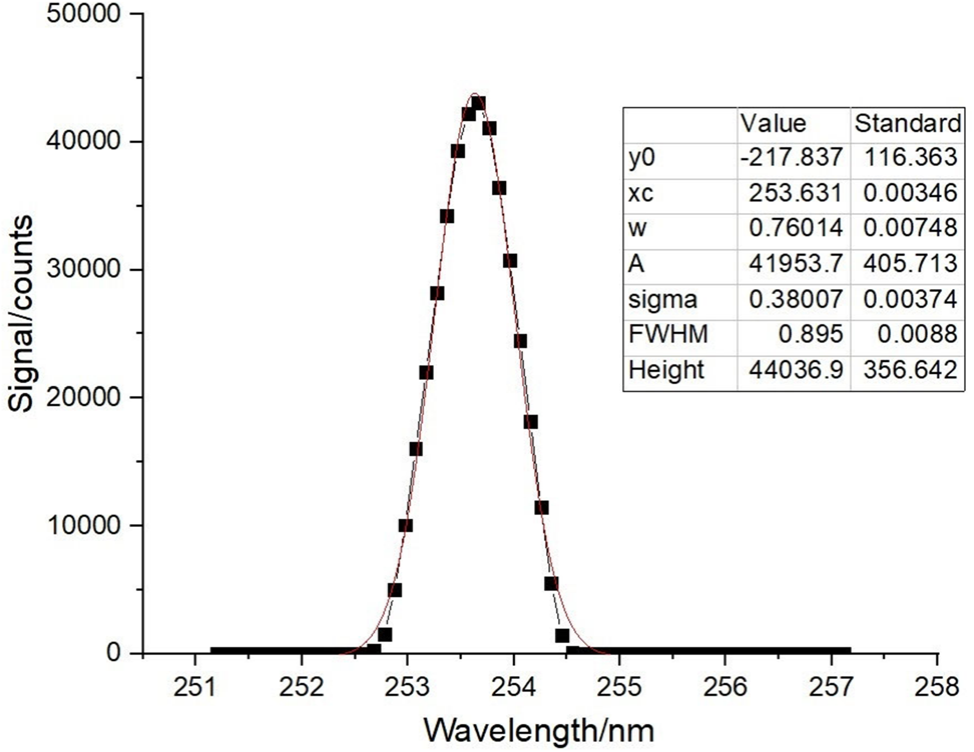
Spectral response and Gaussian fitting curve of mercury lamp at 253 nm.

**Figure 11:**
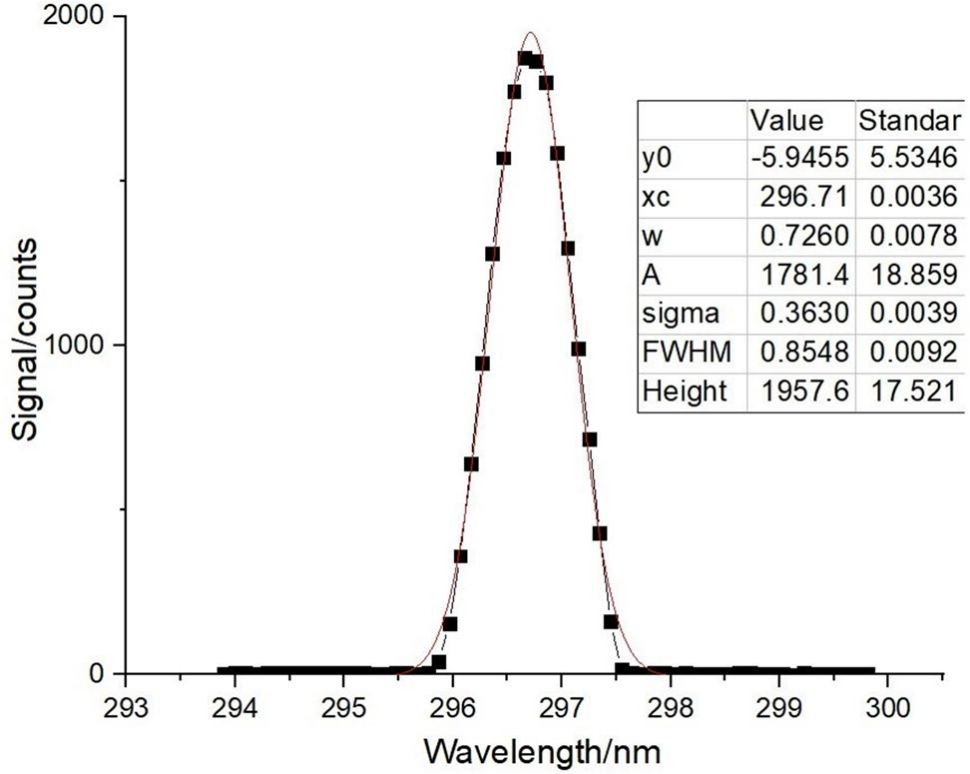
Spectral response and Gaussian fitting curve of mercury lamp at 296 nm.

Control the scanning mechanism according to the method in Section of Control method of wavelength scanning mechanism, and calibrate the spectrum according to the flow and data processing method in Section of Spectral calibration. The proposed method is verified. For the wavelength scanning mechanism, the characteristic spectral lines of mercury lamp at 184 nm, 253 nm and 296 nm are taken, and each spectral line is repeatedly measured for 5 times. The measurement results of any one time are selected for Gaussian fitting, and the original spectral curve and Gaussian fitting results are shown in Figs. 9, 10 and 11.

The central wavelength and spectral resolution of the spectral curve can be directly obtained by all the Gaussian fitting of the curve. The wavelength repeatability and wavelength calibration accuracy are calculated by the processing of the central wavelength of each curve, as shown in Table 2.

**Table 2:** Measurement results of wavelength repeatability and wavelength calibration accuracy.

Number of times	Central wavelength/nm		
1	184.898	253.632	296.713
2	184.887	253.629	296.710
3	184.888	253.627	296.712
4	184.881	253.626	296.710
5	184.882	253.626	296.709
Average value/nm	184.887	253.628	296.711
Standard wavelength/nm	184.891	253.652	296.728
Wavelength repeatability/nm	0.006	0.002	0.002
Wavelength calibration accuracy/nm	0.004	0.024	0.017

### Test results of turntable mechanism

In order to verify the method of controlling stepping motor of turntable mechanism proposed in this paper, deuterium lamp is used as light source. We run the hole position of the turntable mechanism to the plate 01 position (28 steps in clockwise direction), measure 142 times at 240 nm, repeat the test for 5 times, and the measured signal strength stability results are shown in Table 3.

**Table 3:** Signal strength stability.

Number of times	Average signal strength/counts	Average value of overall signal strength/counts	Stability (%)
1	5448.521127	5443.659155	0.089
2	5449.183099		0.101
3	5443.830986		0.003
4	5439.915493		0.068
5	5436.8507		0.125

## Discussion

It can be seen from the test results in Table 2 that the method of controlling the stepping motor of wavelength scanning mechanism proposed in this paper can meet the requirements of wavelength repeatability less than 0.01 nm and calibration accuracy less than 0.05 nm.

It can be seen from Table 3 that the signal intensity stability of deuterium lamp output at the same hole position is less than 0.2%, which meets the requirements of spectrometer.

## Conclusions

Aiming at the design of solar irradiance spectrometer without encoder, a high precision control method of stepping motor is proposed in this paper. The experimental results show that the method can control the stepping motor back to the starting position with high precision, and control the stepping motor running accurately. It meets the requirements of wavelength repeatability less than 0.01 nm, wavelength calibration accuracy less than 0.05 nm, and calibration accuracy stability less than 0.2%. At the same time, it reduces the volume and weight, and realizes the miniaturization of the system.

## Abbreviations

SUSIM: Solar ultraviolet spectral irradiance monitor
SOLSTICE: Solar-stellar irradiance comparison experiment
SIM: Spectral irradiance monitor
SOLSPEC: Solar spectrum
